# Powdery mildew caused by *Erysiphe corylacearum*: An emerging problem on hazelnut in Italy

**DOI:** 10.1371/journal.pone.0301941

**Published:** 2024-05-28

**Authors:** Slavica Matić, Andrea G. Caruso, Chiara D’Errico, Camilla Sacco Botto, Emanuela Noris, Vojislav Trkulja, Stefano Panno, Salvatore Davino, Marco Moizio

**Affiliations:** 1 Institute for Sustainable Plant Protection, National Research Council, Turin, Italy; 2 Department of Agricultural, Food and Forest Sciences, University of Palermo, Palermo, Italy; 3 Agricultural Institute of Republic of Srpska, Banja Luka, Bosnia and Herzegovina; 4 SAGEA Centro di Saggio s.r.l., Castagnito d’Alba (CN), Italy; Tocklai Tea Research Institute, INDIA

## Abstract

*Erysiphe corylacearum* has recently been reported in northern Italy (Piedmont) and other European countries as the causal agent of a new emerging powdery mildew on hazelnut. This disease is much more dangerous than the common hazelnut powdery mildew caused by *Phyllactinia guttata* as it significantly reduces yield and quality of hazelnuts. This study aimed to perform morphological and molecular characterization of the fungal isolates from powdery mildew-infected plants in the Piedmont Italian region. Additionally, genetic diversity studies and pathogenicity tests were conducted. Thirty-six fungal isolates originating from symptomatic hazelnut plants exhibiting specific powdery mildew symptoms on the superior leaf side were identified morphologically as *E*. *corylacearum*. Single- and multilocus sequence typing of five loci (ITS, *rpb2*, *CaM*, *GAPDH* and *GS*) assigned all isolates as *E*. *corylacearum*. Multilocus and *GAPDH* phylogenetic studies resulted in the most efficient characterization of *E*. *corylacearum*. Studied fungal isolates were able to cause new emerging powdery mildew disease by fulfilling Koch’s postulates. The emergence of powdery mildew disease in Italy revealed the *E*. *corylacearum* subgrouping, population expansion, and high nucleotide similarity with other recently identified *E*. *corylacearum* hazelnut isolates. To contain this harmful disease and inhibit the fungus spread into new geographical zones, it will be necessary to implement more rigorous monitoring in neighboring hazelnut plantations near infected hazelnuts, use sustainable fungicides and search for new biocontrol agents.

## Introduction

The powdery mildew fungi are obligate, biotrophic plant pathogens belonging to the Erysiphales order of the Ascomycota phylum [1]. Recently, they have been increasing in Europe due to the spread of novel powdery mildews into new geographic areas accompanied by new species assignment and taxonomic splitting [2, 3].

*Erysiphe corylacearum* is one of the alien pathogens affecting hazelnut (*Corylus avellana* L.; common European hazelnut) in the Middle East and East-Central and Southern Europe. This pathogen has a negative impact on fruit production and safety (U. Braun & S. Takam.), and is the causal agent of the new emerging hazelnut powdery mildew disease [4].

*E*. *corylacearum* has been reported for the first time on hazelnut in Turkey [4]. Before that, it was documented only on other *Corylus* species in Asia, such as Asian hazel (*Corylus heterophylla* Fisch. ex Trautv.), Japanese hazel (*Corylus sieboldiana* Blume) or in America on beaked hazel (*Corylus cornuta* Marshall) [5–7]. After Turkey, it further spread on hazelnut from the Middle East i.e. Iran [8] to East Europe (Ukraine [9], Romania [10]), Central Europe (Austria [11], Hungary [12], Germany [13], Slovenia [14], Switzerland [15]), and Southern Europe (Italy [16] and Spain [17]). Regarding Italy, it was reported for the first time in the Piedmont region in 2020 [16, 18].

This fungal pathogen is much more dangerous than *Phyllactinia guttata* (Wallr.: Fr), the causal agent of the so-called ’common’ hazelnut powdery mildew [19], due to its impact on both the yield and the hazelnut plants life. Symptoms of *P*. *guttata* are visible mainly on the lower surface of the leaf during late summer and have minimal impact on hazelnuts, requiring little or no treatment. On the contrary, *E*. *corylacearum* is particularly harmful to hazelnut cultivation, causing significant damage, that results in a strong reduction in both yield and quality of hazelnuts, especially when associated with water stress [4]. The plants affected by the new powdery mildew show symptoms of a white patina on the upper surface of the leaves; in the case of an intensive attack, the symptoms also occur on young shoots, bracts, and fruits. Powdery mildew lesions turn gradually from yellow to brown colour. In susceptible cultivars, leaves dry out and fruits fall prematurely, resulting in high yield losses [4].

Currently, there are limited curative chemical interventions against *E*. *corylacearum* (e.g. treatment with triazole fungicide Revysion). Farmers may rely on the use of preventive control methods such as the use of sulphur-based fungicides (efficient also against *P*. *corylicola* and gall mites) and sustainable agronomic practices (removal and destruction of the infected leaves and other symptomatic plant material, balanced fertilization, and irrigation).

Currently, there are no available data on the impact of this emerging disease on hazelnuts in Italy. Considering the heavy impact of *E*. *corylacearum* on hazelnut crop in countries where it is already well established, there is a strong risk that this pathogen may profoundly compromise hazelnut production in Piedmont, one of the major Italian hazelnut producers, and that it will spread to other cultivation areas in Italy. The objective of the present study was to characterize the causal agent of the new hazelnut powdery mildew disease found in the Piedmont region. Both morphological observation and single- and multi-locus phylogenetic analyses were performed together with pathogenicity tests to fulfill Koch’s postulates. Furthermore, the pathogen geographical distribution in Piedmont was studied.

## Materials and methods

### Field survey

**Fig 1:**
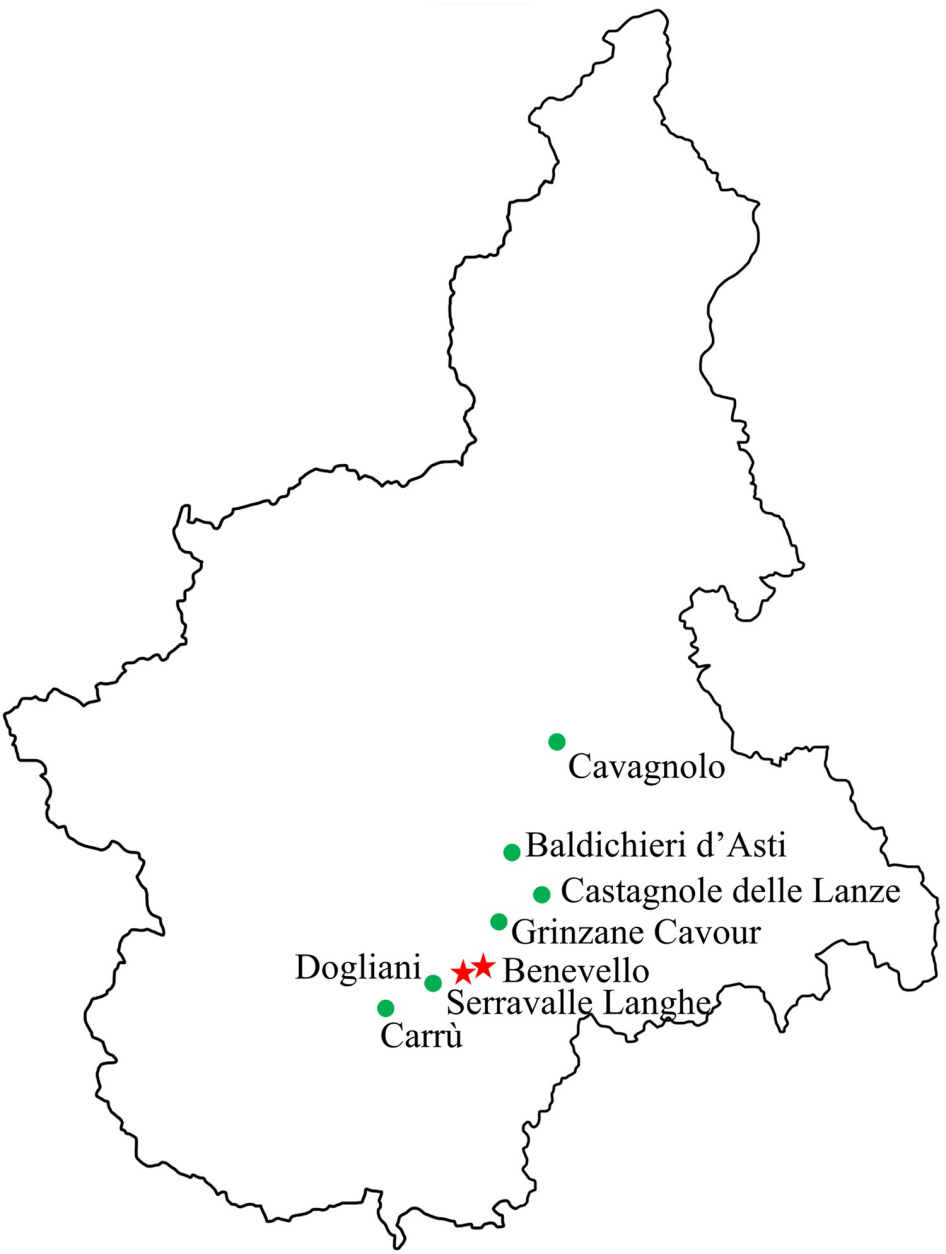
Map of the Piedmont region (North-Western Italy) showing the presence of emerging fungus *Erysiphe corylacearum*, the causal agent of powdery mildew, recently introduced in Italy. The site of samples collection indicated by a star are those where *E*. *corylacearum* is detected during 2022 field-survey, while locations marked by a dot correspond to the fungus-free zones.

A field survey was performed in the Piedmont region (North-Western Italy). During the late spring-summer period (June-August) of 2022, eight hazelnut orchards planted with the ‘Tonda Gentile delle Langhe’ (Trilobata) cultivar have been observed for *E*. *corylacearum* symptoms in eight different locations of Torino, Cuneo and Asti provinces (Fig 1). Leaves showed the characteristic symptoms of new powdery mildew associated with *E*. *corylacearum* and they appeared as round, white, powdery spots visible mainly on the upper surface of the leaves and gradually increased in size. The leaves also showed symptoms of common powdery mildew, visible mostly on the lower part of the leaf. Symptomatic leaves were collected and transported in paper bags to the laboratory for morphological observations and molecular analyses.

### Morphological characterization

A total of 160 isolates were collected from eight locations and examined for their morphological characteristics. Each isolate was collected from a single powdery mildew spot from the upper or lower leaf surface of a single hazelnut plant.

To make anatomical observations, hyphae, conidiophores, conidia, and chasmothecia from a single powdery mildew spot were stripped off from the leaf surface with a clean needle and mounted in water. The remaining portion of the sample (isolate) was conserved and used for molecular study if necessary. Slides were observed under a Leica DM750 biological microscope (Leica Microsystems GmbH, Wetzlar, Germany) equipped with a Leica EC4 camera (Leica Microsystems GmbH, Wetzlar, Germany). Anatomical features were measured using ScopeImage 9.0 with 40× objective. Microscopic observations of all isolates were carried out, determining the shape and size of conidia, asci, ascospores, chasmothecia and appendages. The obtained results were compared with the species descriptions reported by Braun and Cook [7] and Bradshaw et al. [20]. Based on morphological observations, 36 random fungal isolates were selected and used in subsequent molecular analyses.

### DNA extraction, PCR and sequencing

Mycelia containing both conidia and chasmothecia of a single powdery mildew spot were scraped from the leaf surface and used for DNA extraction with 5% Chelex (Bio-Rad, Hercules, California, USA), as described previously by Hirata and Takamatsu [21]. DNA extracts were used directly for PCR amplification of the following DNA regions: internal transcribed spacer (ITS), RNA polymerase second largest subunit (*rpb2*), calmodulin (*CaM*), glyceraldehyde-3-phosphate dehydrogenase (*GAPDH*), and glutamine synthetase (*GS*). The ITS region was amplified using either primers ITS1 and ITS4 [22] or ITS1 and PM6 [23]. The other four genes were amplified as described by Bradshaw et al. [24], using the following primers: PmRpb2_4 and PMRpb2_6R (*rpb2*), PMCAM1 and PMCAM4R (*CaM*), PMGAPDH1 and PMGAPDH3R (*GAPDH*), and GSPM2 and GSPM3R (*GS*). PCR products were purified using a QIAquick PCR purification kit (Qiagen, Hilden, Germany), in accordance with manufacturer’s instructions and sequenced in both directions at the BMR Genomics Centre (Padua, Italy). The obtained sequences were deposited in the NCBI GenBank database under the following accession numbers: OQ917083- OQ917118 for ITS, OR126170- OR126205 for *rpb2*, OQ995105- OQ995140 for *CaM*, OR105713- OR105735 for *GAPDH*, and OR126217- OR126241 for *GS* (Table 1).

**Table 1:** List of *Erysiphe corylacearum* isolates used for the molecular identification and of their corresponding accession numbers.

Isolate	Site of collection	ITS	*rpb2*	*CaM*	*GAPDH*	*GS*
1_PdIT	Serravalle Langhe (Cuneo province, Piedmont region)	OQ917083	OR126170	OQ995105	OR105700	OR126206
2_PdIT		OQ917084	OR126171	OQ995106	OR105701	OR126207
3_PdIT		OQ917085	OR126172	OQ995107	OR105702	OR126208
4_PdIT		OQ917086	OR126173	OQ995108	OR105703	OR126209
5_PdIT		OQ917087	OR126174	OQ995109	OR105704	OR126210
6_PdIT		OQ917088	OR126175	OQ995110	OR105705	OR126211
7_PdIT		OQ917089	OR126176	OQ995111	OR105706	OR126212
8_PdIT		OQ917090	OR126177	OQ995112	OR105707	OR126213
9_PdIT		OQ917091	OR126178	OQ995113	OR105708	OR126214
10_PdIT		OQ917092	OR126179	OQ995114	OR105709	OR126215
11_PdIT		OQ917093	OR126180	OQ995115	OR105710	OR126216
38_PdIT		OQ917108	OR126195	OQ995130	OR105725	OR126231
39_PdIT		OQ917109	OR126196	OQ995131	OR105726	OR126232
40_PdIT		OQ917110	OR126197	OQ995132	OR105727	OR126233
41_PdIT		OQ917111	OR126198	OQ995133	OR105728	OR126234
42_PdIT		OQ917112	OR126199	OQ995134	OR105729	OR126235
43_PdIT		OQ917113	OR126200	OQ995135	OR105730	OR126236
44_PdIT		OQ917114	OR126201	OQ995136	OR105731	OR126237
45_PdIT		OQ917115	OR126202	OQ995137	OR105732	OR126238
46_PdIT		OQ917116	OR126203	OQ995138	OR105733	OR126239
47_PdIT		OQ917117	OR126204	OQ995139	OR105734	OR126240
48_PdIT		OQ917118	OR126205	OQ995140	OR105735	OR126241
23_PdIT	Benevello (Cuneo province, Piedmont region)	OQ917094	OR126183	OQ995116	OR105713	OR126217
24_PdIT		OQ917095	OR126185	OQ995117	OR105715	OR126218
25_PdIT		OQ917096	OR126184	OQ995118	OR105714	OR126219
26_PdIT		OQ917097	OR126186	OQ995119	OR105716	OR126220
27_PdIT		OQ917098	OR126181	OQ995120	OR105711	OR126221
28_PdIT		OQ917099	OR126187	OQ995121	OR105717	OR126222
29_PdIT		OQ917100	OR126188	OQ995122	OR105718	OR126223
30_PdIT		OQ917101	OR126189	OQ995123	OR105719	OR126224
31_PdIT		OQ917102	OR126190	OQ995124	OR105720	OR126225
32_PdIT		OQ917103	OR126191	OQ995125	OR105721	OR126226
33_PdIT		OQ917104	OR126182	OQ995126	OR105712	OR126227
34_PdIT		OQ917105	OR126192	OQ995127	OR105722	OR126228
35_PdIT		OQ917106	OR126193	OQ995128	OR105723	OR126229
36_PdIT		OQ917107	OR126194	OQ995129	OR105724	OR126230

### Sequence analyses

The ITS sequences of all 36 isolates obtained were aligned with the reference *Erysiphe* sequences available in the GenBank database, using the BLAST software package (www.ncbi.nlm.nih.gov). Subsequently, Maximum Likelihood (ML) phylogenetic analyses were performed on both single and concatenated sequences of five loci (ITS, *rpb2*, *CaM*, *GAPDH* and *GS*). Concatenated phylogenetic analyses were also performed on the basis of Bayesian inference (BI) using Geneious Prime v. 2020.1.2 (Biomatters Ltd., New Zealand). Heating parameter in BI phylogenetic analyses was adjusted to 0.2 by sampling the trees at every 1000 generations. BI analyses were terminated when the mean standard deviation of the split frequencies was less than 0.01. The ITS phylogenetic analyses were carried out including 26 reference sequences of *E*. *corylacearum* and 11 reference sequences of close Erysiphe phylogenetic species infecting *Corylus* spp., such as *E*. *pseudocorylacearum*, *E*. *cornutae*, *E*. *coryli-americanae*, *E*. *corylicola*, and *E*. *syringae* [20]. Other single and multilocus-sequence phylogenetic analyses included only the 36 isolates from this study, as gene sequences of reference *E*. *corylacearum* isolates were not available. In all phylogenetic analyses, *E*. *necator* (FH00941202) sequences were used as the outgroup. Concatenated dataset of five loci included a total of 2,397 bp. The best-fit nucleotide model of each dataset for a ML analysis was determined using Findmodel (http://www.hiv.lanl.gov/content/sequence/findmodel/findmodel.html): JC: Jukes-Cantor for ITS, TrN: Tamura-Nei plus Gamma for *CaM* and *GAPDH* and concatenated tree, and HKY: Hasegawa-Kishino-Yano for *rpb2* and *GS*. The ML analyses were carried out with MEGA 11 software [25]. The most appropriate nucleotide model of each partition was identified by means of MrModeltest v.2.3 [26] and used for BI analyses.

DNA polymorphism parameters (haplotype diversity, nucleotide diversity, number of polymorphic sites, mutations and nucleotide differences) were determined by means of DNA Sequence Polymorphism v. 6 software [27].

Tajima’s D, Fu’s and Li’s D, and Fu’s and Li’s F tests determined departures from the null hypothesis of neutral evolution. Significant values of these tests can indicate the presence of population changes such as change in size, population expansion, population contraction, and population subdivision [28, 29].

### Pathogenicity assays

Two-years old healthy hazelnut plants of ‘Tonda Gentile delle Langhe’ cv. were used for the pathogenicity assays. Plants were maintained in 20 L plastic pots containing a sterilized mixture of 80% peat and 20% perlite in a greenhouse.

Three plants were inoculated with powdery mildew on three randomly selected branches, using 10 μL of a conidial suspension (1 × 10^5^ spores mL^−1^) obtained by washing conidial spores from the field-collected heavily infected leaves with powdery mildew symptoms on the upper leaf side.

Plants were then maintained under growth chamber conditions: 25 °C (day), 19°C (night), 12-h photoperiod and once daily watering. Three non-inoculated plants were kept away from inoculated ones under the same conditions in a greenhouse and served as control plants.

Re-isolations were performed from leaves artificially inoculated with powdery mildew fungus and from control plants. Macro- and micro-morphological characteristics of the hyphae, conidiophores and conidia stripped off from leaves of the inoculated plants were observed. Specific symptoms of powdery mildew and disease severity were evaluated at three time-points starting 10 days post-inoculation (dpi) (10, 15 and 20 dpi).

Disease severity (DS) as the percentage of visually infected leaf area was evaluated by averaging three inoculated plants. Statistical analyses for the pathogenicity assay were performed using PAST v. 4.03 software [30]. Differences in DS between different time-points were analyzed by a one-way ANOVA, and in case of significant results (P < 0.05), the Turkey HSD was used for mean separation.

## Results

### Field survey

Out of eight hazelnut orchards inspected, typical *E*. *corylacearum* symptoms were observed in two orchards located in the Benevello and Serravalle Langhe areas of the Cuneo province. Symptoms associated with *E*. *corylacearum* were not found in the remaining sites in the provinces Torino and Asti (Fig 1). In these two provinces, only symptoms specific to common powdery mildew caused by *P*. *guttata* were observed.

**Fig 2:**
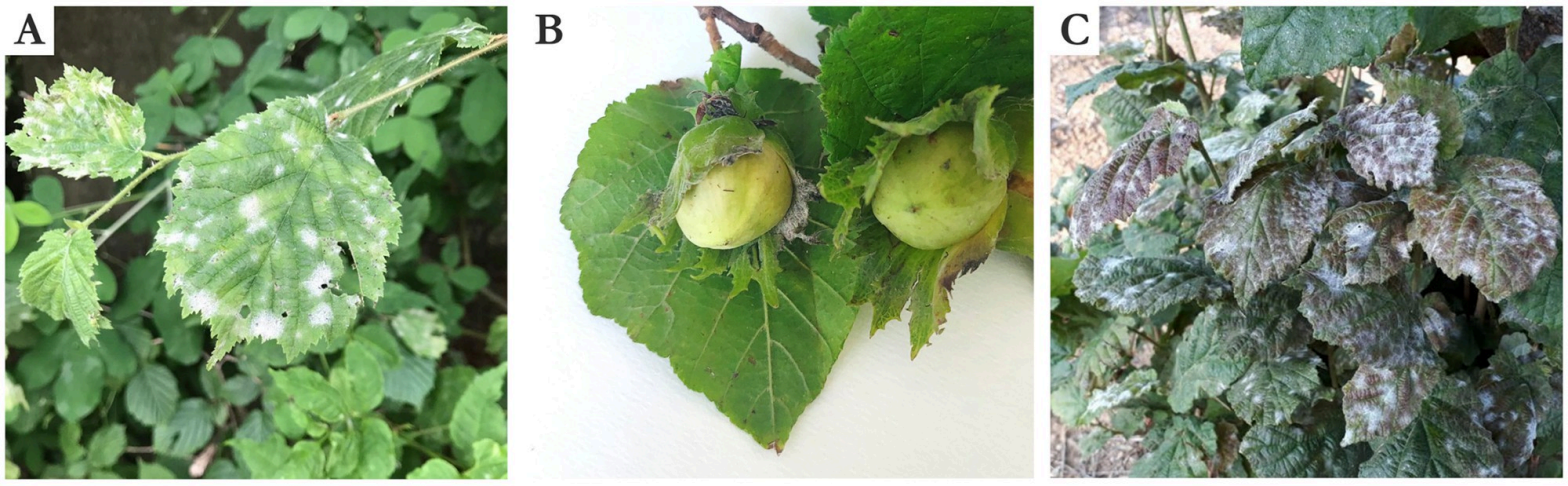
Symptoms of *Erysiphe corylacearum* on hazelnut leaves. (A) 0.5–1 cm circular, powdery white spots and pale to yellow leaf areas on upper leaf surface; (B) White to grayish mycelium growth on fruit bracts; (C) Progressive growth of the powdery spots and turning the leaf color to purple-bronze in late infection stage.

Symptoms caused by *E*. *corylacearum* have been observed from late spring through the summer. These symptoms consisted of small circular, powdery white spots on the adaxial leaf surface, measuring a few millimeters in size, with pale to chlorotic leaf areas. Lesions of yellow to brown color corresponding to the upper white spots were observed on the lower leaf surface. Powdery spots progressively increased to 1–1.5 cm in size (Fig 2A), and also occurred on young branches and fruit bracts at later inspections (Fig 2B). Infected leaves tended to a purple-bronze color (Fig 2C). Finally, heavily infected leaves wilted prematurely, and affected fruits dried and occasionally fell.

### Morphological characterization

Morphological observations of 160 samples from eight locations confirmed the presence of *E*. *corylacearum* only in samples from powdery mildew spots from the upper leaf side in two orchards; Benevello and Serravalle Langhe. All other samples collected from the remaining six locations where common powdery mildew was observed did not show the presence of *E*. *corylacearum*. In these samples, the presence of *P*. *guttata* was only observed (data not shown). These morphological results confirmed the results of the field survey and indicated that *E*. *corylacearum* is predominantly found on the ‘upper’ powdery spots of the hazelnut leaves, and *P*. *guttata* is found on the ‘lower’ ones.

**Fig 3:**
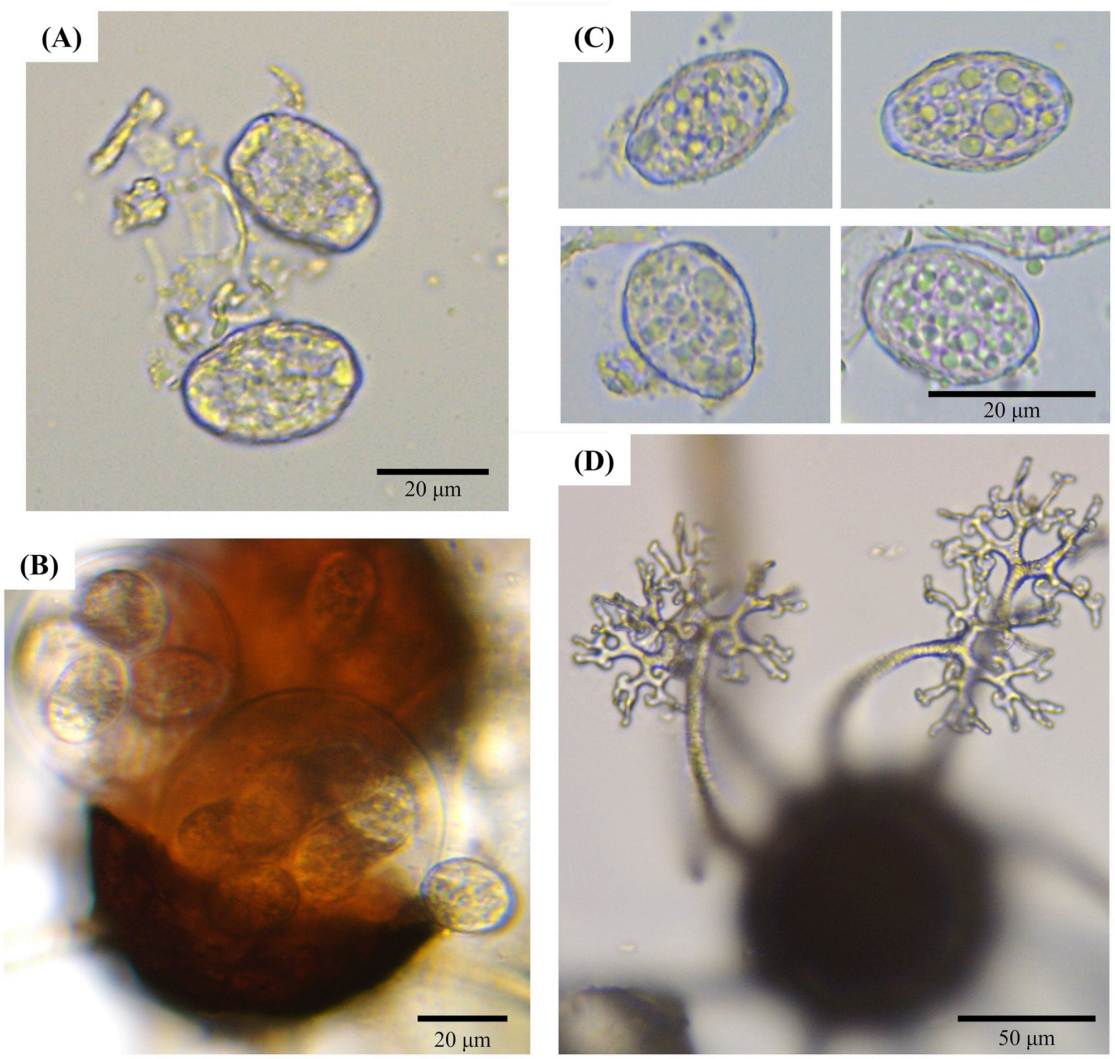
Morphological characteristics of *Erysiphe corylacearum* isolates from hazelnut in this study. (A) Conidia; (B) Chasmothecium realising the asci with ascospores; (C) Ascospores; (D) Chasmothecium and its appendages. Scale bar = 20 μm (A-C), 50 μm (D).

Microscopic observation of 36 selected *E*. *corylacearum* samples mainly revealed a mycelium on the upper side of leaves, with the following characteristics: 2–5 μm wide, white, branched, hyaline, septate, and thin-walled. The size of conidiophores was around 69 ± 4 × 7.1 ± 1.2 μm. Size and shape of conidia were similar among all isolates, showing an ellipsoid to ovoid shape, hyaline, with size of 32.3 ± 0.8 × 20.4 ± 0.7 μm, and they were produced individually on the conidiophores (Fig 3).

Chasmothecia were scattered to gregarious on the lower side of leaves, with a diameter of 88 ± 10 μm; 6 to 14 hyaline appendages for each chasmothecium. Appendages were long about the chasmothecial diameter, equatorial, with a thick base and thinner upper part, and with regularly dichotomously branched apices and curved tips. Each chasmothecium contained 3–5 asci of obovoid to ellipsoid shape with a size of 44 ± 1 × 33.2 ± 0.8 μm, containing 5–7 hyaline ascospores of ellipsoid to ovoid shape with a size of 20.0 ± 0.7 × 12.4 ± 0.3 μm (Fig 3). Following the key for the identification to the species of *Erysiphe* on *Corylus* hosts [20], all studied isolates were identified as *E*. *corylacearum*.

Based on morphological observations, 36 *E*. *corylacearum* isolates were selected and used in subsequent molecular analyses. Among them, 22 isolates originated from Serravalle Langhe location and 14 isolates from Benevello location (Table 1).

### Molecular identification and phylogenetic analyses

The alignment of 36 ITS sequences from this study with the reference *Erysiphe* spp. sequences available in the GenBank database showed the highest identity (99.6–100%) with *E*. *corylacearum*, while a lower identity (98.9–99.2%) was observed with *E*. *pseudocorylacearum*, the closest phylogenetic relative of *E*. *corylacearum* [20].

**Fig 4:**
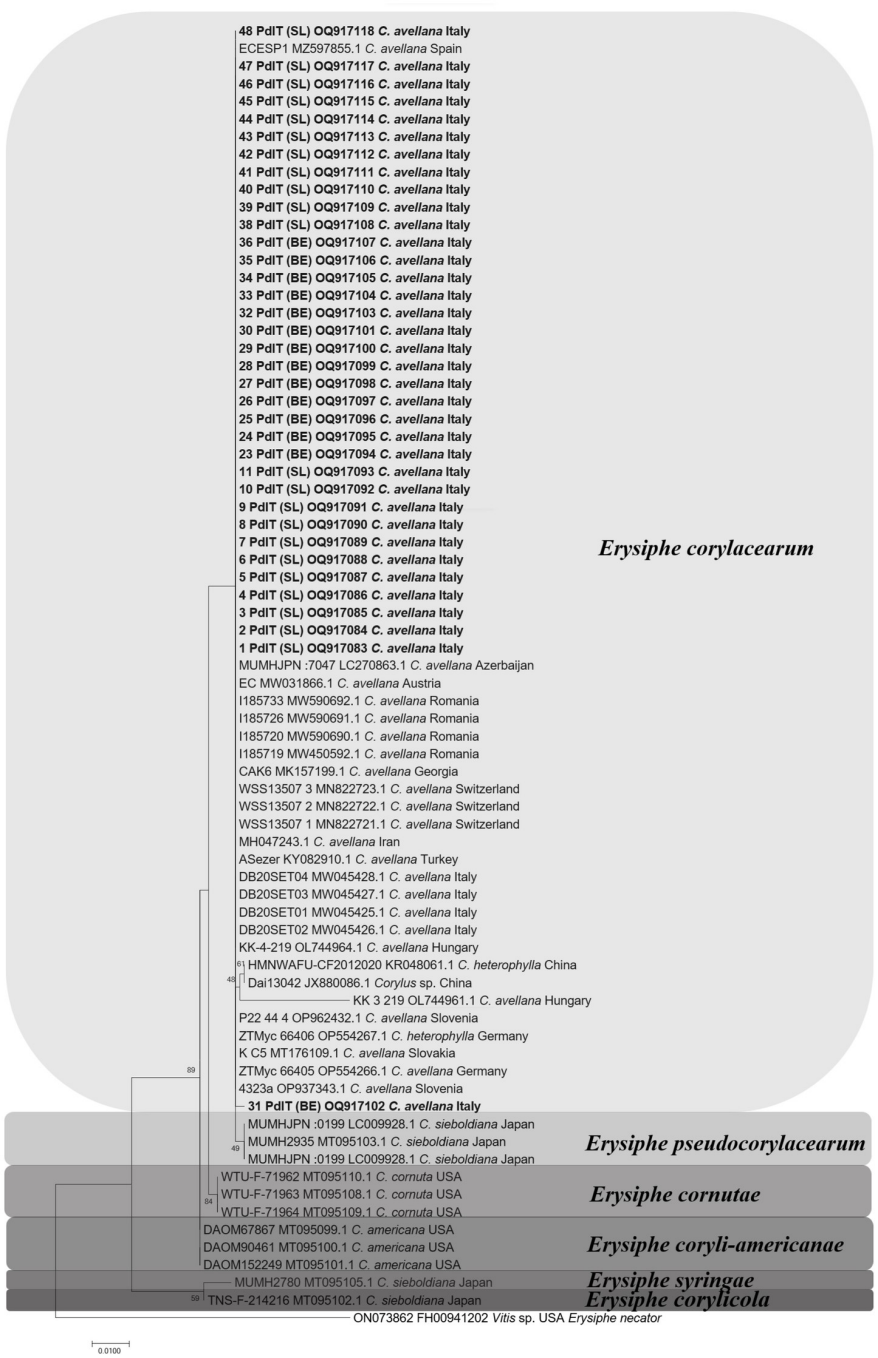
Phylogenetic analysis of *Erysiphe corylacearum* isolates from hazelnut obtained on the basis of ITS inferred from Maximum Likelihood analysis. The values at the nodes indicate bootstrap support values based on 1000 replicates. For each isolate, the isolate name, the Accession Number, the host affiliation, and the geographic origin are shown. The isolates of *E*. *corylacearum* from this study are shown in bold and their collection site is shown in parentheses; (BE) = Benevello, (SL) = Serravalle Langhe. The isolate FH00941202 of *Erysiphe necator* was used as outgroup.

Phylogenetic analyses based on ITS sequences of the studied isolates and of 11 reference isolates of six *Erysiphe* species grouped all 36 isolates with *E*. *corylacearum* (Fig 4).

All studied isolates clustered in the same cluster with *E*. *corylacearum* from Italy, Spain, Austria, Switzerland, Germany, Slovakia, Slovenia, Hungary, Romania, Azerbaijan, Turkey, Georgia, and Iran. Within this major group, only *E*. *corylacearum* Chinese isolates HMNWAFU-CF2012020 and Dai13042 from Asian hazel, and Hungarian KK_3_219 isolate from hazelnut formed a separate subcluster.

Phylogenetic analyses carried out for the single-locus sequences (*rpb2*, *CaM* and *GAPDH*) permitted a further distinction of the studied isolates in distinct *E*. *corylacearum* subclusters, while the GS analysis grouped all studied isolates together as the ITS region (S1 Fig).

**Fig 5:**
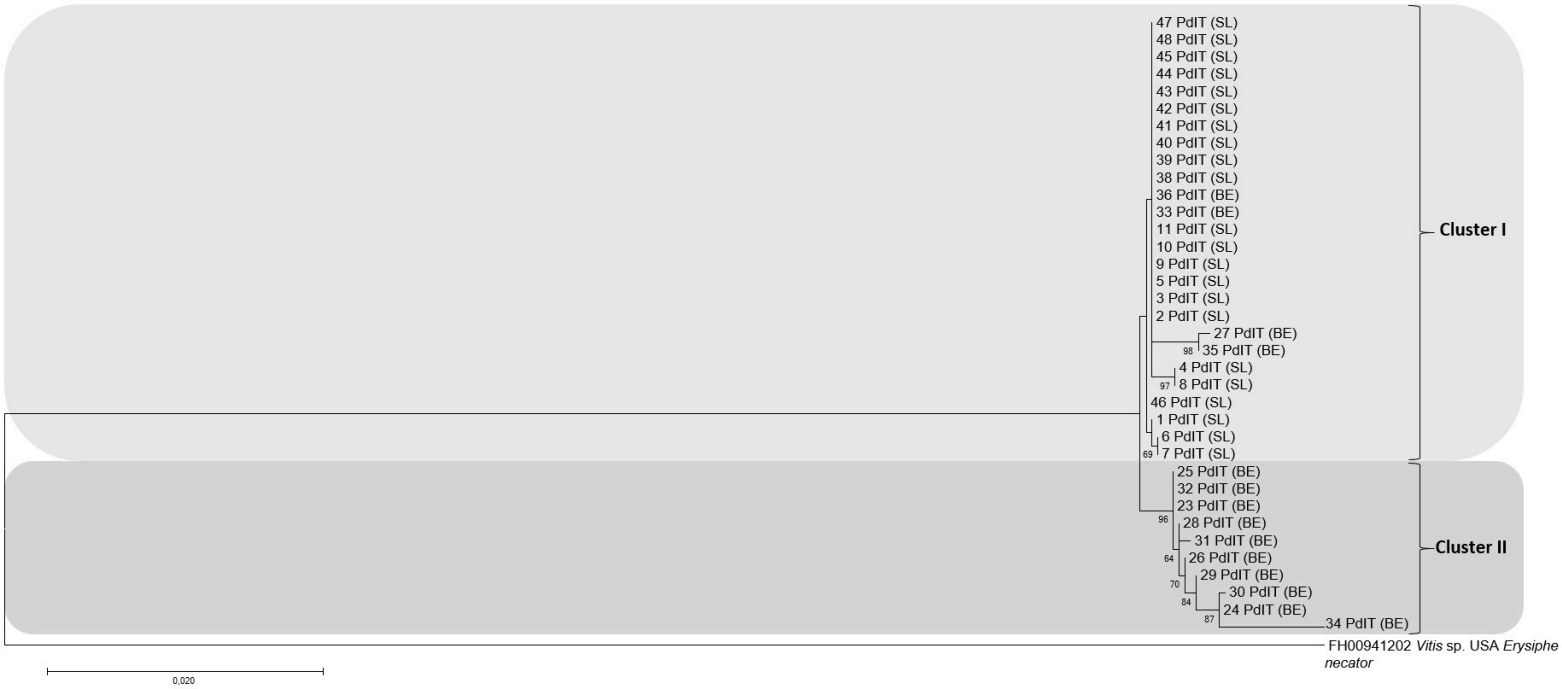
Phylogram based on ITS, *rpb2*, *CaM*, *GAPDH* and *GS* sequences of the studied *Erysiphe corylacearum* isolates. The site of their collection is shown in parentheses; (BE) = Benevello, (SL) = Serravalle Langhe. The concatenated phylogenetic tree was obtained by Maximum Likelihood analysis using the Tamura-Nei plus Gamma model. The isolate FH00941202 of *Erysiphe necator* was used as outgroup.

Finally, concatenated ML phylogenetic analyses based on all five loci (ITS, *rpb2*, *CaM*, *GAPDH* and *GS*) were performed and the obtained results allowed a more robust differentiation of the studied isolates discerning 4 different subclusters when compared to single loci (Fig 5). The first subcluster included predominantly isolates from the Serravalle Langhe orchard with two isolates from the Benevello orchard, while the other three subclusters contained isolates exclusively from one orchard, either Serravalle Langhe or Benevello. Concatenated BI phylogenetic analyzes confirmed the results of grouping and subgrouping of the studied isolates obtained by ML analyses (S2 Fig).

Overall, the ITS region revealed 4 haplotypes within Italian and reference isolates with a low degree of haplotype diversity (Hd) (0.125), as well as a low degree of nucleotide diversity per site (π) (0.00120) (S1 Table). Tajima’s D, Fu’s and Li’s D, and Fu’s and Li’s F tests were significantly negative which may indicate the population expansion or selective processes. Two independent studies, including this one and one performed by Mezzalama et al. [18] found higher DNA polymorphism among reference isolates originating from eleven countries compared to the Italian isolates originating from Piedmont. The haplotypes identified in this study were mainly associated with singleton variable sites. On the other hand, no InDel haplotypes were found in the examined region by the DNASP software, and they were not associated with insertion/deletion mutations.

### Pathogenicity assays

**Fig 6:**
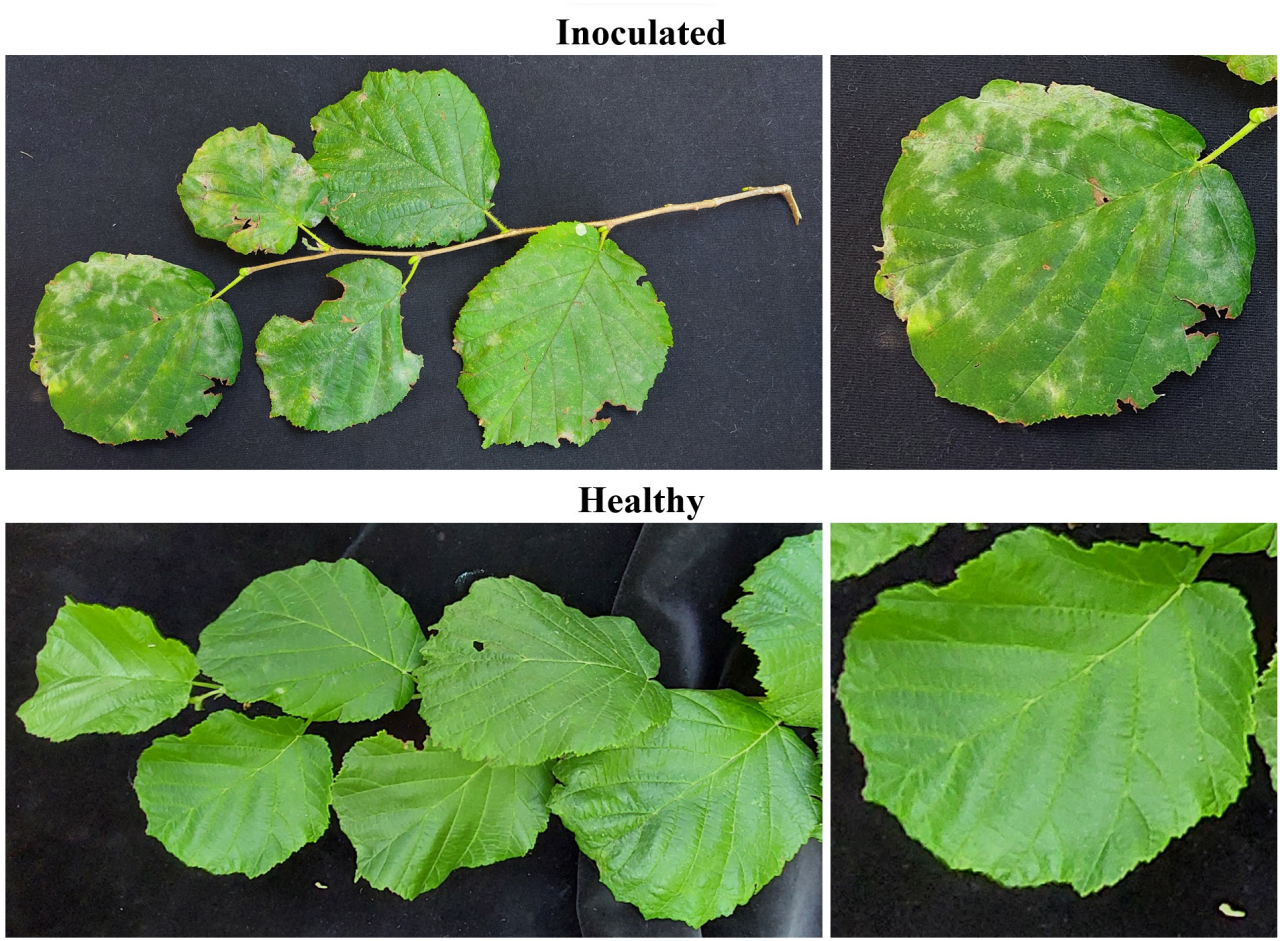
Pathogenicity test with *Erysiphe corylacearum*: (a) an inoculated and (b) a non-inoculated hazelnut plant. Powdery mildew symptoms observed 20 days post-inoculation.

The first symptoms became visible on inoculated hazelnut branches at 10 dpi, consisting of tiny white powdery spots on the upper leaf surface. White spots gradually extended along with the onset of chlorotic leaf areas. On the lower leaf side, yellow to brown areas corresponding to the upper powdery spots were discerned. Inoculated hazelnut plants showed typical powdery mildew symptoms at 15–20 dpi, while control plants were asymptomatic (Fig 6).

During the development of the powdery mildew disease, *E*. *colyracearum* specific symptoms were observed during all three observation periods (10,15 and 20 dpi), and DS increased gradually during the time course of the disease. Symptoms between all three-time course periods were statistically significant and the highest incidence of the disease was observed at 20 dpi when DS reached about 40%. (S3 Fig).

## Discussion

A recent taxonomic study on powdery mildew disease agents caused by *Erysiphe* species on *Corylus* hosts in the world includes six species; *E*. *cornutae*, *E*. *corylacearum*, *E*. *coryli-americanae*, *E*. *corylicola*, *E*. *pseudocorylacearum*, and *E*. *syringae* [20]. Beside them, *P*. *guttata* is a globally widespread powdery mildew agent on hazelnut, of no particular economic concern for hazelnut growers [4, 7, 19, 31].

In this study, 36 new strains, originating from one geographic zone (Cuneo province in the Piedmont region) isolated from hazelnut plants showing powdery mildew disease symptoms on the upper leaf side, were identified as *E*. *corylacearum*. We demonstrated that these strains were the causal agent of the new powdery mildew disease. On the other hand, surveyed hazelnut orchards in Turin and Asti provinces with typical symptoms of common powdery mildew did not show the presence of *E*. *corylacearum*, and only the presence of *P*. *guttata* was found in them.

The results of our study are of particular importance because *E*. *corylacearum* is increasingly damaging hazelnut cultivation not only in the Piedmont region, but also throughout the European Union, where it is considered as an emerging pathogen [32]. Originally found only in Asia and America on other hazelnut species (Asian, Japanese and beaked hazelnut), it was able to spread in less than a decade to hazelnut trees from Turkey and Iran to Eastern, Central and Southern Europe, including Italy [7]. Moreover, it will probably expand further into new geographic regions.

All the isolates examined in this study were identified morphologically as *E*. *corylacearum* following the identification key for the *Erysiphe* species on *Corylus* hosts [20]. However, this key follows the strict host specialization and, due to a possible host range expansion of *Erysiphe* spp., confirmatory molecular analyses were required for the correct identification of the isolates.

Molecular characterization confirmed the morphological identification in this study. Given that ITS-based identification is not always sufficient for the precise identification of powdery mildew species [33] and considering that the majority of previous studies on *E*. *corylacearum* were based exclusively on the ITS molecular characterization [8, 10–13, 15, 18], we undertook identification using also other loci [24]. The GS phylogenetic analyses grouped all 36 isolates with *E*. *corylacearum*, in a way similar to the ITS marker. However, when the *rpb2*, *CaM* and *GAPDH* loci were used in phylogenetic analyses, it was possible to better differentiate studied isolates separating them into two distinct *E*. *corylacearum* subclusters. This confirmed the usefulness of the markers *rpb2*, *CaM* and *GAPDH* reported by results of Bradshaw and coworkers [24] allowing an improved broad and fine scale phylogenetic analyses of powdery mildew.

Finally, concatenated ML and BI phylogenetic analyses based on all five loci (ITS, *rpb2*, *CaM*, *GAPDH* and *GS*) permitted the most robust phylogenetic scaling of *E*. *corylacearum*, as already reported in similar studies of fungal identification [24, 34–36]. This analysis enabled not only a differentiation into two separate clusters, but also made possible to separate the major cluster into four different subclusters. The most similar results with the combined phylogenetic analysis were achieved with the *GAPDH* marker, which indicates its potential and usefulness in future phylogenetic analyses of *E*. *corylacearum*. High-resolution genetic typing approach of MLST in this study reflected the results of previous MLST works of hazelnut powdery mildews [24], but for more comprehensive concatenated phylogenetic studies, it will be necessary the multi-locus sequencing of *E*. *corylacearum* from other countries.

The recent emergence of *E*. *corylacearum* in Italy and throughout many European countries may be associated with the accidental import of infected common hazelnut propagating material from Asia, which is susceptible to this powdery mildew. Another possibility is that it was present in latent form on Japanese hazel that was imported in Europe at the beginning of the twentieth century [37] or on some other exotic hazelnuts. Since host jumps and/or host range expansions may happen in powdery mildews [38], it might be that *E*. *corylacearum* started to spread from exotic on other well-established *Corylus* spp. in Europe such as common and Turkish hazelnut (*Corylus colurna*). One additional hypothesis is that *E*. *corylacearum* gradually spread from its initial infection zone (Turkey and Iran) towards to Europe via wind. Obligate biotrophs such as powdery mildews are known for their long-distance dispersal habit. They may be transported as structures able to cause an infection up to ≥ 1000 km from initial sites, threating the plant health and yield [39] if they are supported by the production of huge numbers of wind-dispersed spores from one plant to another, essential for the survival of these obligately biotrophic fungi [40, 41]. Moreover, powdery mildews asci show ‘explosive discharge’, known to move in nature with speed exceeding 30 m/s (or 100 km/h) [42].

Regarding the neutrality tests in this study and taking in consideration that all *E*. *corylacearum* (studied and reference) isolates showed negative values for Fu and Li’s D and Fu and Li’s F statistics, the expansion of population size or population selection may be suggested similar to reports for other emergent fungi [43–45]. However, negative but not significant values of neutrality tests among the known Italian *E*. *corylacearum* isolates may indicate that the fungus recently arrived, but that further expansion throughout the country has not yet initiated.

It will be interesting to have more *E*. *corylacearum* gene sequences available from other countries and the whole fungal genome in the GenBank database. This would allow to search for adaptive founder events which frequently occur in powdery mildew genomes, such as single nucleotide variations, transposition events, and genome rearrangements. These events might be also one of the possible drivers of adaptation of powdery mildews, including *E*. *corylacearum*, to new plant hosts and environmental conditions [46, 47].

## Conclusion

Conclusively, this study has shown that the emerging hazelnut powdery mildew in Northern Italy is caused by *E*. *corylacearum*. Wind-spread, infected planting material, susceptibility of ‘Tonda Gentile delle Langhe’, the major cultivar of hazelnut grown in Piedmont, to *E*. *corylacearum* are potential key aspects that should be considered in efficient management of new emerging plant diseases. Moreover, more surveys in neighboring hazelnut plantations near infected plantations, the use of sustainable fungicides and the search for new biocontrol agents will be necessary for an efficient containment of this disease. These initiatives, together with the concomitant search of resistant cultivars and the use of fungicides with different modes of action, may contribute to a successful management of this new emerging and dangerous powdery mildew disease.

## Supporting information

S1 Fig Phylogenetic analyses of 36 *Erysiphe corylacearum* isolates based on single sequences from *rpb2* (a), *CaM* (b), *GAPDH* (c), and *GS* (d). Each phylogenetic tree was obtained by Maximum Likelihood analysis. Reference strains included in the phylogenetic analyses of 5 loci are indicated in bold. Bootstrap values of less than 50% are not presented. The tree was rooted to *Erysiphe necator* (FH00941202).
(TIF)

S2 Fig Consensus phylogram based on ITS, *rpb2*, *CaM*, *GAPDH* and *GS* sequences of the studied *Erysiphe corylacearum* isolates. The concatenated phylogenetic tree was obtained by Bayesian analysis using the GTR substitution model. The isolate FH00941202 of *Erysiphe necator* was used as outgroup.
(TIF)

S3 Fig Powdery mildew disease severity on artificially infected hazelnut plants at 10, 15 and 20 days post-inoculation (dpi). The means and standard deviations of 9 replicates were shown.
(TIF)

S1 Table Molecular parameters of the isolates of *Erysiphe corylacearum* obtained from partial ITS sequences. (DOCX)
