# Supplementary figures and images for: Powdery mildew caused by Erysiphe corylacearum: An emerging problem on hazelnut in Italy
Source: PLoS One. 2024 May 28;19(5):e0301941. doi: 10.1371/journal.pone.0301941 (PMC11132447; doi:10.1371/journal.pone.0301941)

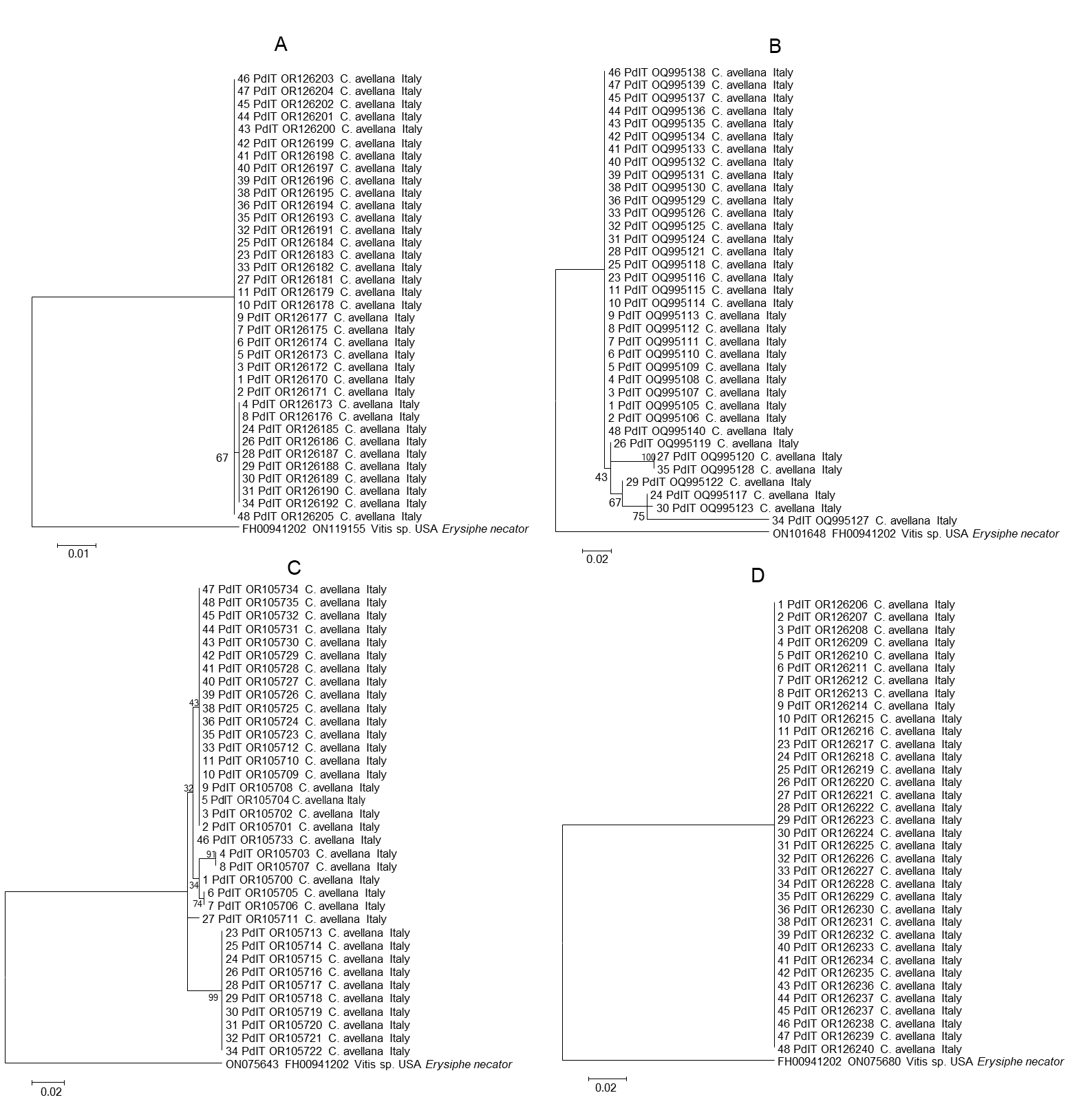

Supplement: S1 Fig — Phylogenetic analyses of 36 Erysiphe corylacearum isolates based on single sequences from rpb2 (a), CaM (b), GAPDH (c), and GS (d). Each phylogenetic tree was obtained by Maximum Likelihood analysis. Reference strains included in the phylogenetic analyses of 5 loci are indicated in bold. Bootstrap values of less than 50% are not presented. The tree was rooted to Erysiphe necator (FH00941202). (TIF) [file pone.0301941.s001.tif]

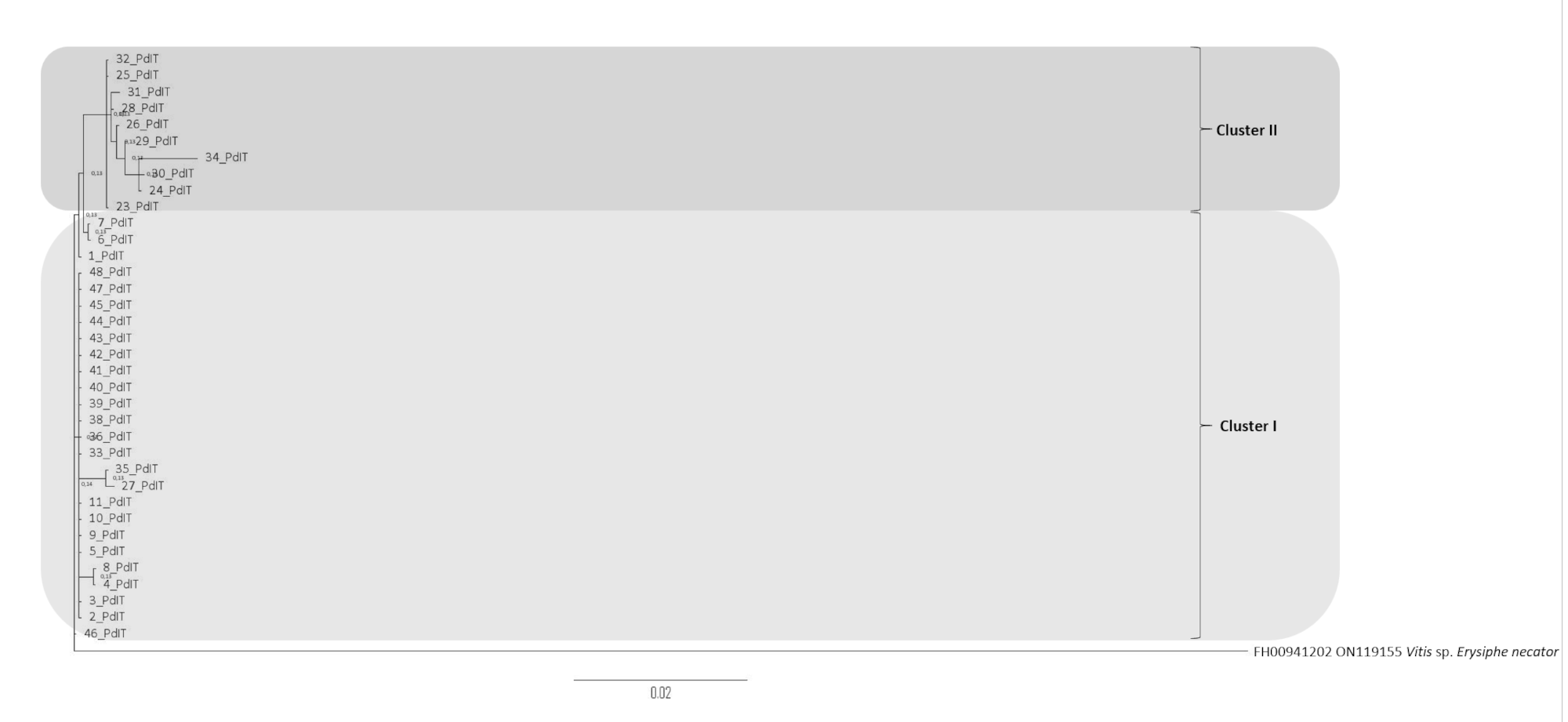

Supplement: S2 Fig — The concatenated phylogenetic tree was obtained by Bayesian analysis using the GTR substitution model. The isolate FH00941202 of Erysiphe necator was used as outgroup. (TIF) [file pone.0301941.s002.tif]

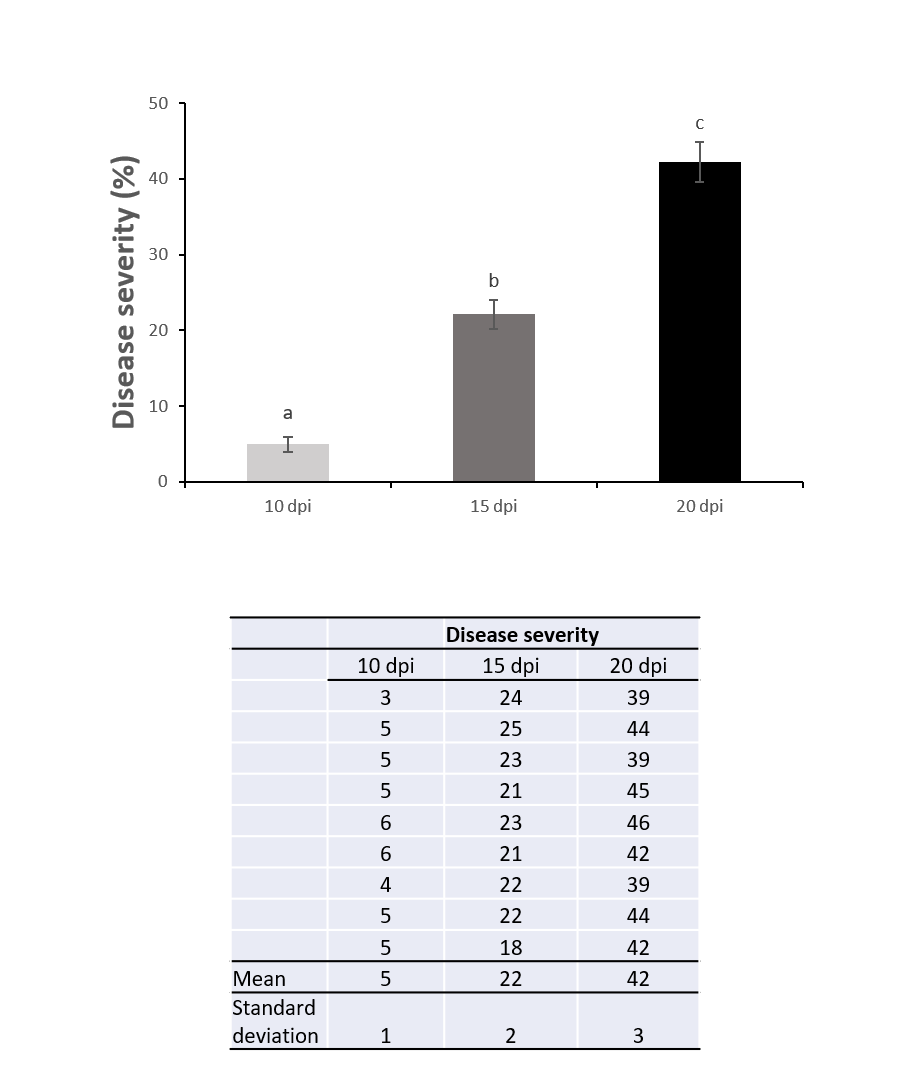

Supplement: S3 Fig — The means and standard deviations of 9 replicates were shown. (TIF) [file pone.0301941.s003.tif]
