# Supplementary material for: Powdery mildew caused by Erysiphe corylacearum: An emerging problem on hazelnut in Italy
Source: PLoS One. 2024 May 28;19(5):e0301941. doi: 10.1371/journal.pone.0301941 (PMC11132447; doi:10.1371/journal.pone.0301941)
Supplement: S1 Table — (DOCX) [file pone.0301941.s004.docx]

**Supplementary Table 1.**  Molecular parameters of the isolates of *Erysiphe corylacearum* obtained from partial ITS sequences.

| **Polymorphism parameter** | **All** | **Italian** | **Reference** |
| --- | --- | --- | --- |
| Number of isolates | 62 | 40 | 22 |
| Analysed region | 1-683 | 1-683 | 1-683 |
| Number of haplotypes, H | 4 | 2 | 3 |
| Number of polymorphic sites, S | 12 | 2 | 11 |
| Number of mutations, Eta | 13 | 2 | 12 |
| Singleton variable sites | 12 | 2 | 10 |
| Haplotype diversity, *h* | 0.125 | 0.05 | 0.255 |
| Nucleotide diversity, π | 0.00120 | 0.00015 | 0,00311 |
| θ (S) | 0.00120 | 0.00072 | 0,00821 |
| θ (π) | 0.00696 | 0.00015 | 0.00311 |
| Average number of nucleotide differences, *k* | 0.449 | 0.100 | 1.16450 |
| Tajima's D | -2,42393 (P < 0.01) | -1,48662 (P>0.10) | -2.26432 (P < 0.01) |
| Fu and Li's D | -4,94345 (P < 0.02) | -2.42724 (P>0.05) | -3.20280( P < 0.02) |
| Fu and Li's F | -4,83100 (P < 0.02) | -2.49579 (P>0.05) | -3.40305 (P < 0.02) |
